# Embodiment into a robot increases its acceptability

**DOI:** 10.1038/s41598-019-46528-7

**Published:** 2019-07-12

**Authors:** J. Ventre-Dominey, G. Gibert, M. Bosse-Platiere, A. Farnè, P. F. Dominey, F. Pavani

**Affiliations:** 1 Inserm, Stem Cell and Brain Research Institute U1208, 69500 Bron, France; 2 Univ Lyon, Université Claude Bernard Lyon 1, Lyon, France; 3 Integrative Multisensory Perception Action & Cognition Team (ImpAct), Lyon Neuroscience Research Center (CRNL), 69003 Lyon, France; 4 Hospices Civils de Lyon, Neuro-Immersion, 69003 Lyon, France; 5 Center for Mind/Brain Sciences (CIMeC), University of Trento, Rovereto, Italy; 6 Department of Psychology and Cognitive Sciences, University of Trento, Rovereto, Italy

**Keywords:** Biotechnology, Cognitive neuroscience

## Abstract

Recent studies have shown how embodiment induced by multisensory bodily interactions between individuals can positively change social attitudes (closeness, empathy, racial biases). Here we use a simple neuroscience-inspired procedure to beam our human subjects into one of two distinct robots and demonstrate how this can readily increase acceptability and social closeness to that robot. Participants wore a Head Mounted Display tracking their head movements and displaying the 3D visual scene taken from the eyes of a robot which was positioned in front of a mirror and piloted by the subjects’ head movements. As a result, participants saw themselves as a robot. When participant’ and robot’s head movements were correlated, participants felt that they were incorporated into the robot with a sense of agency. Critically, the robot they embodied was judged more likeable and socially closer. Remarkably, we found that the beaming experience with correlated head movements and corresponding sensation of embodiment and social proximity, was independent of robots’ humanoid’s appearance. These findings not only reveal the ease of body-swapping, via visual-motor synchrony, into robots that do not share any clear human resemblance, but they may also pave a new way to make our future robotic helpers socially acceptable.

## Introduction

A major challenge when building robots that operate in everyday contexts is the definition of those features that can make the robot socially accepted by humans. Science fiction narratives as well as cognitive neuroscience research have inspired technological development based on similarity of perceived features. Anthropomorphic details have thus been introduced into the body and face of robots and avatars, including colour changes in the face area to simulate flushes linked to emotional reactions^1^. However, two lines of cognitive neuroscience research exploiting the cognitive mechanisms underlying embodiment suggest that a different and yet unexplored approach to robot social acceptance may also be possible. The first line of the research shows the importance of 1st-person-perspective and agency in the embodiment of single body-parts, other humans or virtual reality avatars^2–5^. For example, by manipulating the visual perspective in conjunction with multisensory information correlated between a subject and a mannequin, researchers have induced illusions of owning another body instead of the one’s own^3,6^. The second line of results reveals that the effects of embodiment can go well beyond bodily illusions and extend to self- and social-perception^7–9^. Thus, when a subject facing a partner is touched on the face in synchrony and at the same location as the partner’s face, an illusion of enfacement can occur suggesting that features of other’s identity can be incorporated in the representation of self^10^. Such a change in self-face recognition contingent on visuo-tactile facial stimulation has been extended to social and conceptual representations. For example, Peck *et al*.^11^ demonstrated in a virtual reality environment that the embodiment of a light-skinned individual in a dark-skinned virtual body decreases that individual’s implicit racial bias.

Here we tested for the first time the possibility that social acceptability of robots (including non-humanoids) can be achieved through their embodiment. We developed a simple procedure that allows observers to beam into the robot’s body, (see ref.^12^), promoting its embodiment and, ultimately, its social acceptance. In this study, the terms “to beam” or “beaming” make reference to a form of physical transportation to a distant location (and borrowed from the 1960’s American television series Star Trek). Here, beaming refers to a method of embodiment in a robot. Via a 3D stereo head-mounted display, the subject sees through the eyes of the robot, and via a motion capture system, the subject’s head movements control the robot in real time. Thus the subject can direct their gaze in a remote environment and see the corresponding dynamic visual scene in 3D^13^.

We conducted two experiments that contrasted beaming in correlated (synchronous) vs. static (Exp 1) and correlated vs. uncorrelated (Exp 2) human and robot head movements. We measured social acceptance (Likeability and Closeness) and embodiment (Enfacement, Location and Agency) scores for each robot before and after the beaming experience, using standardised questionnaires. The experiments were performed with two robots distinct in their structures: the robot iCub, having a humanoid structure, and the robot Reeti that resembles a cartoon character. Critically, both robots had mobile eyes and heads. By using two different robots, we wanted to assess whether robot embodiment and ensuing social acceptance would depend on the robot having or not a closer resemblance to the structure and morphology of the human body. Accordingly, in order to determine the respective effects of robot morphology, humanoid vs non-humanoid (R-Type) and of the robot state, static vs. correlated vs uncorrelated (R-State), behavioral responses were compared between R-Type and R-State.

These novel results reveal that our simple and rapid beaming procedure can produce systematic changes into the observer’s social attitude towards robots. By taking a different stance towards acceptability (i.e. not based on appearance), built on embodiment research, we have provided proof-of-concept that beaming into robots can overcome the major challenge of making robots socially acceptable.

## Results

### Experiment 1

#### Likeability measurement

**Figure 1:**
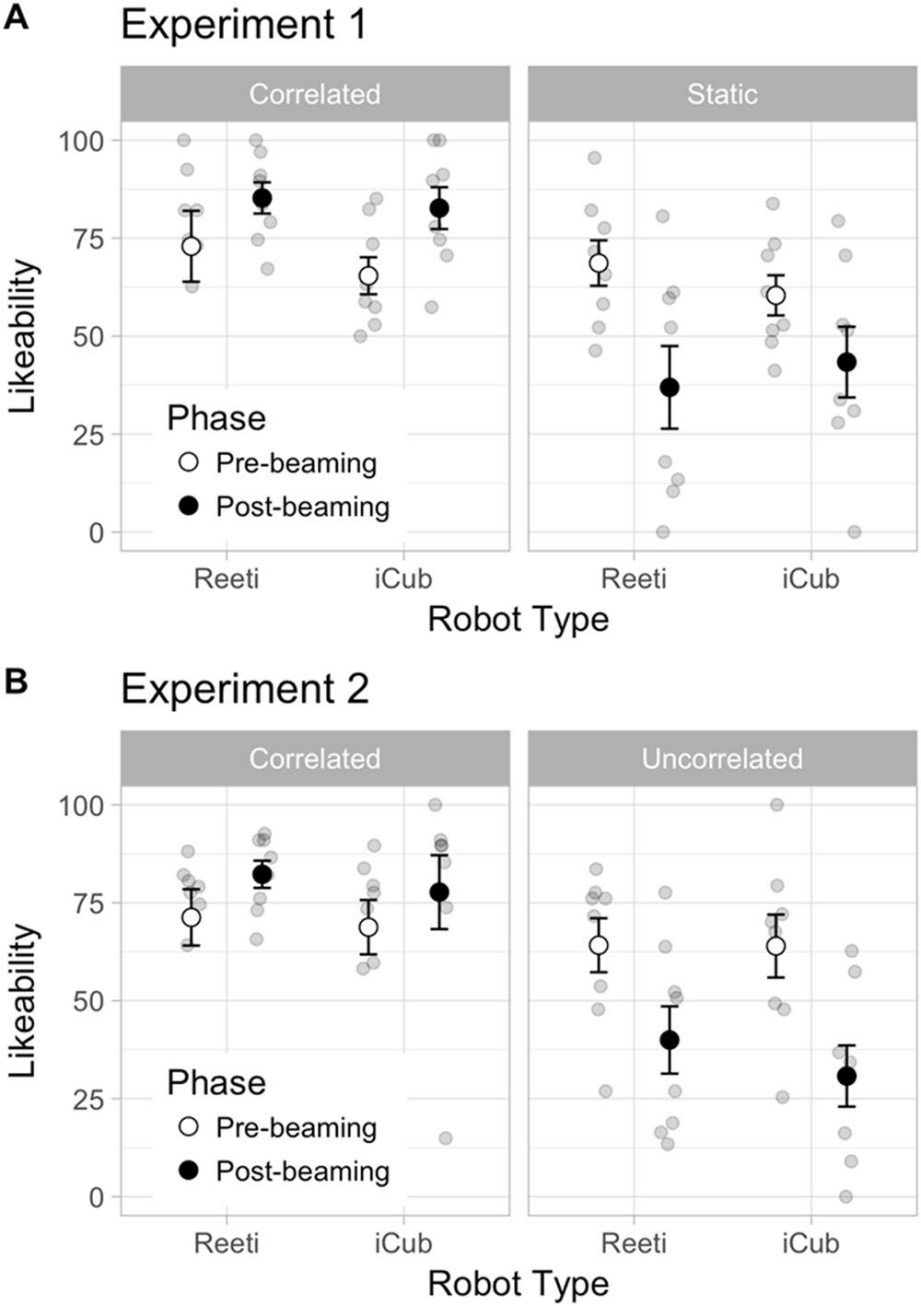
Likeability result: Means plots showing the distribution of the likeability scores measured in Experiment 1 (**A**) and Experiment 2 (**B**), before (Pre-beaming) and after (Post-beaming) beaming inside Reeti and iCub robots. Note the general increased likeability scores in the Correlated as compared to Static and Uncorrelated conditions. The Likeability score can reverse after beaming as a robot can become more likeable after Correlated and less likeable after Static and Uncorrelated conditions. Points: Individual data; Circles: Means; Bars: Standard errors.

As shown in Fig. 1A, the Likeability rate was independent of the factor R-Type (F(1,14) = 0.11, p > 0.25; partial η2 = 0.008). In contrast, the Likeability was strongly dependent on the Beaming experience that is the Pre-beaming and the Post-beaming tested respectively before and after the two beaming sessions. Thus, when comparing the Beaming experiences (Pre vs Post) and Robot States (Static vs Correlated), we found a significant effect of the factor R-State (F(1,14) = 16.75, p = 0.001; partial η2 = 0.55) and a significant Beaming Experience x R-State interaction (F(1,14) = 18,50, p < 0.001; partial η2 = 0.57), independently of the Type of Robot (Beaming Experience x R-State x R-Type interaction (F(1,14) = 1.16, p > 0.25; partial η2 = 0.077). The Likeability score decreased after the Static as compared to the Correlated state (p < 0.001) and to the Pre Beaming Experience (p = 0.012). Interestingly as illustrated in Fig. 1A, the Likeability score could benefit from the Correlated State of the Robot.

#### IOS test: interactions self and other

**Figure 2:**
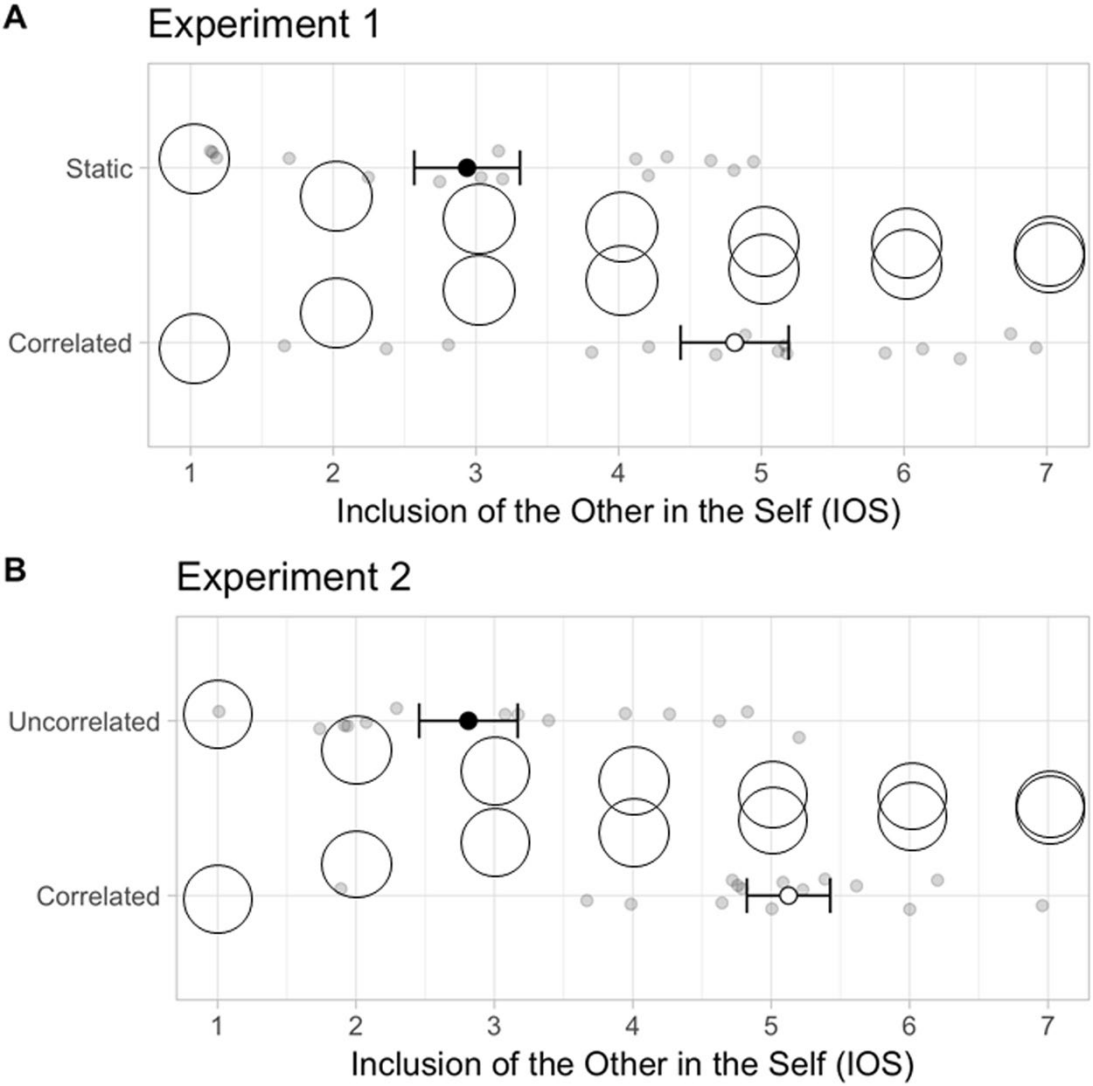
Closeness result: Closeness rating (IOS score) on a 1 to 7 scale estimating the degree of overlap between self and other as the overlap between the circles (Inclusion of Other in the Self Scale = IOS test). The plots represent the IOS scores measured after beaming in Static and Correlated conditions in Experiment 1 (A: upper panel) and in Uncorrelated and Correlated conditions in Experiment 2 (B: lower panel). Note the general increase of closeness in the Correlated as compared to Static and Uncorrelated conditions. Points: Individual data; Small Circles: Means; Bars: Standard errors.

As shown in Fig. 2A, the Closeness rate measured by the IOS test increased in a Correlated as compared to a Static beaming for both robots. Indeed, the IOS score was significantly depending on the factor R-State (F(1,14) = 50.8, p < 0.001; partial η^2^ = 0.78**)** with no relation with the factor R-Type (R-State x R-Type Interaction: F(1,14**) = **0.22, p > 0.25; partial η^2^ = 0.016). The Closeness rate was related to the type of human-robot interactions with a highly significant increase of IOS score in Correlated as compared to Static state (p < 0.001).

#### Test of embodiment

**Figure 3:**
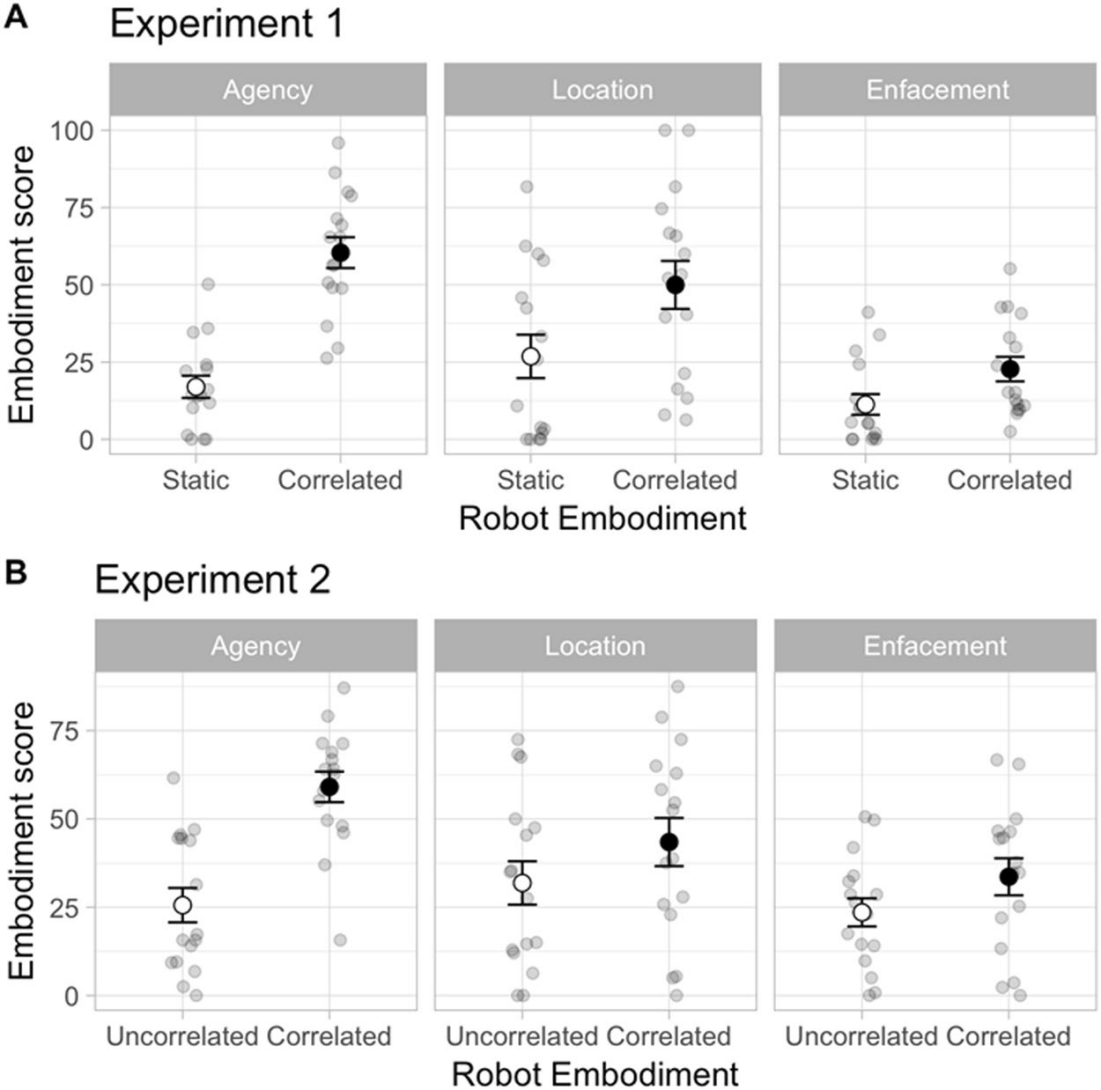
Embodiment results: Mean values of the embodiment scores for the enfacement, location and agency items as measured after beaming inside the robots in Experiment 1 (A: upper panel) and Experiment 2 (B: lower panel). Note the general increase in location and agency scores in the Correlated as compared to Static and Uncorrelated conditions. Points: Individual data; Circles: Means; Bars: Standard errors.

In Fig. 3A, we observe that the Embodiment increased in the correlated vs. static beaming conditions, but that this varies with the different categories (enfacement, location, agency). Thus, the Embodiment score was significantly depending on the factor R-State (F(1, 14) = 59.00, p < 0.001; partial η^2^ = 0.81) and the Embodiment Category (F(2,28) = 15.80, p < 0.001: partial η^2^ = 0.53). In addition, the effects of the Robot State on the Embodiment score significantly varied with the Category (R-State x Category interactions (F(2,28) = 12.02, p < 0.001; partial η^2^ = 0.46) and with the Type of Robot (R-Type x R-State x Category interactions (F(2, 28) = 4.83, p = 0.016; partial η^2^ = 0.26). While the enfacement score was independent of the factor R-State (p > 0.25), the location and the agency scores significantly increased in the Correlated as compared to Static states respectively at p = 0.004 and p < 0.001 (Fig. 3A). Furthermore, in Correlated state, the enfacement score was significantly different from the location (p < 0.001) and agency (p < 0.001) scores. In contrast, no significant difference was found between the location and agency scores (p > 0.25).

Interestingly, we found significant correlations – in the Correlated state, between the agency and the Likeability score (R = 0.67, p = 0.005), the agency and the IOS score (R = 0.64, p = 0.007) and between the location and the Likeability score (R = 0.71, p = 0.002) and – in the Static state, only between the agency and the IOS score (R = 0.69, p = 0.003) and between the Likeability and IOS scores (R = 0.82, p < 0.001).

#### Inferences on inner states

No correlation was found between the Subject responses and the Robot 3^rd^ perspective responses for any states of the Robot.

### Discussion

These results suggest that beaming into a robot moving in correlated manner with a subject’s head induces a significant sensation of embodiment, essentially of the location and agency categories as well as an increased likeability and closeness towards the robot. These effects are not found by beaming into a robot which remains static despite the subject’s movements.

In order to investigate the contribution of the head movements on the illusory changes after beaming, we performed a second experiment in which we compared the effects of correlated versus uncorrelated head movements on the social acceptance and embodiment features.

### Experiment 2

#### Likeability measurement

As in Experiment 1, after the beaming experience, we observed that the Likeability increased after correlated beaming, and decreased after uncorrelated beaming for both robots. This is confirmed, as the Likeability score was significantly related to the factor R-State (F(1,14) = 18,37, p < 0.001; partial η^2^ = 0.57), but not to the factor R-Type of Robot (R-State x R-Type Interaction: F(1,14) = 0.49, p > 0.25; partial η^2^ = 0.034). When comparing the different states of the Robots between the beginning (Pre) and the end (Post) of the experiment, we found a significant main effect of Beaming Experience (F(1,14) = 15, p = 0.0017; partial η^2^ = 0.52), and a significant interaction Beaming Experience x R-State (F(1,14) = 8.91, p = 0.01, partial η^2^ = 0.39), independently of the Robot Type (Beaming Experience x R-Type interaction: F(1,14) = 0.51, p > 0.25, partial η^2^ = 0.035; Beaming Experience x R-State x R-Type interaction: F(1,14) = 0.18, p > 0.25; partial η^2^ = 0.013). As shown in Fig. 1B, the Likeability score decreased for both robots after the Uncorrelated state as compared to the Pre state (p = 0.044) and significantly differed between Uncorrelated and Correlated states (p = 0.0015).

#### IOS test: interactions self and other

As shown in Fig. 2B, the IOS score significantly changed with the factor R-State (F(1,14) = 52.37, p < 0.001; partial η^2^ = 0.79) independently of the factor R-Type (R-State x R-Type interaction: F(1,14) = 0.34, p > 0.25; partial η^2^ = 0.024). The IOS score was significantly increased in Correlated as compared to Uncorrelated state (p < 0.001).

#### Test of embodiment

The Embodiment score was significantly depending on the R-State (F(1, 14) = 38.46, p < 0.001; partial η^2^ = 0.73) and the Category (F(2,28) = 5.08, p = 0.013; partial η^2^ = 0.27) as well as their R-State x Category interactions (F(2,28) = 9.20, p < 0.001; partial η^2^ = 0.40). The embodiment score did not change with the R-Type (R-State x R-Type interactions: F(2,28) = 1.59, p > 0.25; partial η^2^ = 0.10; R-State x Category x R-Type interactions: F(2,28) = 0.27, > 0.25; partial η^2^ = 0.019). The Embodiment score was significantly increased for the agency category only in Correlated as compared to Uncorrelated state (p < 0.001). In Correlated state, we found that the enfacement rate was significantly different from the agency rate (p = 0.002). The results obtained with the test of Embodiment are illustrated in Fig. 3B.

In Uncorrelated state, we found that the agency significantly correlated with the Likeability score (R = 0.70, p = 0.002), the IOS score (R = 0.67, p = 0.005) and the enfacement (R = 0.65, p = 0.006). The enfacement was strongly correlated to the Likeability score (R = 0.84, p < 0.001).

#### Inferences on inner states

No correlation was found between the Subject responses and the Robot 3^rd^ perspective responses for any States of the Robot.

### Discussion

These results suggest that the correlation between human and robot head movements constitutes a crucial cue to induce a significant sensation of embodiment, essentially through agency as well as an increased likeability and closeness towards the robot.

## General Discussion

This study demonstrated for the first time that the social acceptance of a robot, in particular the likeability, can be increased with embodiment and decreased with lack of embodiment. This emerged regardless of whether a robot appeared humanoid or not (iCub is designed to appear with a human-like shape; Reeti evokes instead a non-humanoid fiction character). Possible cognitive processes that link body representation and social acceptance will be discussed in the following sections.

### Embodiment and self awareness

Here, embodiment in robots has been induced by generating synchronous exploratory head-movements between the robot and the subject. As the robot is facing a mirror, the subject who sees through the robot’s eyes, perceives him/herself as the robot’s head. As the subject’s and robot’s heads move synchronously, the subject experiences the sensation of controlling the movements of the robot’s head. This mirror synchrony promotes a strong sense of agency over the robot and, while less prominent, a sense of location inside the robot body. These findings are in line with previous observations of bodily illusion showing that congruent movements between real and artificial body-parts, often involving hands, will produce illusory feeling of ownership and agency over the artificial body-part^2,14–16^. While the sense of ownership is strictly depending on congruent posture and temporal synchrony between the movements, the sense of agency has been sometimes reported in incongruent or distant locations^2,14^ and in asynchronous movements^16^. The subjective sensation of embodiment generally occurs with a drift in the perceived location of the real body-part toward the fake one, so-called proprioceptive drift^14^. Similarly in our study, as compared to the static condition a sense of location and agency over the robot occurs both in synchronous/correlated and less so in uncorrelated head movements with the two robots, humanoid (iCub) or non-humanoid (Reeti). However one can ask what is a non-humanoid robot like the one used in our study? Is it comparable to an artefact or to a somewhat body like item? These issues have been previously raised in rubber hand illusion (RHI) experiment by comparing the rate of embodiment into an artificial hand versus a wooden object^17–19^. These authors found that embodiment was not possible in artefacts like a wooden stick even during a synchronous stroking of the subject’s hand and the object. Similarly, out-of-body experiences demonstrate that subjects can feel the sensation to own or to be localized in another body like an avatar or a mannequin but not in a scrambled body^3,20,21^. As a whole, these authors provide evidence that the viewed object has to resemble the body shape to allow for embodiment. Based on these findings, we suggest that even though our non-humanoid robot appeared as a fiction personage, it was made of a trunk (without arms) supporting a head with eyes, nose and mouth, features corresponding to the topology of a biologically physical face. But how far can we induce embodiment in non-humanoid robots? In the current study we initiated this interrogation by comparing humanoid (iCub) and non-humanoid (Reeti) robots with potentially animated facial characters. In this context we observed a null effect on robot-type which indicates that robot-type did not impact on the effect of synchrony and embodiment on acceptability. This is consistent with the interpretation that variability along the humanoid dimension does not necessarily impact the possibility for embodiment and acceptability. In the future, it would be pertinent to investigate the physical limit for a robot that will prevent further embodiment.

In contrast to the subjective sensation of location and agency, subjects experienced no significant sense of enfacement in any beaming conditions. Similar to the ownership sensation over an artificial hand in the Rubber Hand Illusion experiment (RHI), enfacement has often been described following sensory manipulations on the face^8,10,22,23^. Typically, the participant is looking at a face of an unknown subject who is stroked on the same face area as the participant. The congruence and synchrony of the visuo-tactile stimulations elicit an illusion of ownership over the other face. Similarly, Preston *et al*.^6^ described strong feeling of ownership over an artificial body seen in a mirror during simultaneous stroking of both mannequin and participant’s bodies. In our experiment, even though the subject is facing the robot face through a mirror that reinforces self-enfacement, the embodiment is only induced by head movements likely less susceptible to induce a sense of ownership. Such an issue could be further studied in using visuo-tactile stimulation of the faces of participant and robot. In line with our observations, dissociation between ownership and agency has previously been reported during motor RHI experiment. Indeed, while passive movements of a fake hand as compared to a real hand eliminate agency and preserve ownership, the opposite is observed with active movements of the hand placed in an incongruent position^2,16^.

As a whole, our beaming experience of human into a robot reveals a strong effect of embodiment including sense of agency over the robot and a feeling of mislocation of one’s own body inside the robot. In a similar approach of teleportation of a human subject into a standing up robot, several authors^24,25^ demonstrated the subject’s ability to develop a sense of embodiment of the robot’s body while keeping a sense of location in her/his own body (illusion of bilocation). Using Brain Computer Interface (BCI) system to control humanlike android, Alimardini *et al*.^26^ induced embodiment into robot’s hand when the subject imagines a movement (motor imagery task) and watches the robot executing it. The authors showed that the motor imagery skill was better with humanlike hands than with metallic grips^27^. Interestingly, this approach using BCI system suggests that embodiment into a robot is well grounded into visuo-motor processes.

The sensation of embodiment arises from multimodal integration of conflicting bodily signals and in the case of motor driven illusion between motor (efferent copy), vision and proprioceptive signals. When seeing oneself in a mirror as a robot head moving along with one’s own movement, the participant experienced vivid impression to voluntary control the robot movements. Such a feeling of self-produced movement occurs as the predictive sensory copy from the motor command matches the effective sensory feedback that supports the sense of agency over the robotic body. Furthermore, in our study this was observed irrespective of whether the robot appeared humanoid or not. In line with the previous findings using BCI technology, our observation is suggestive of a purely sensori-motor grounded process with internal body remapping driven by confounding visuo-motor information. Such a transformation of internal body representation might interfer with body self-recognition and self-awareness.

### Self awareness, social cognition and robot acceptance

As described above, a number of body manipulations including rubber hand illusion^28,29^, out-of-body experience^3,21,30^, interpersonal multimodal stimulation^7,8,23^ had provided better understanding of the cognitive basis of self-awareness and its plasticity in mental representation (for a review, see also ref.^31^). Interestingly, recent researches in social cognition provide evidence for a continuum in recognition of self and others as the perception of bodily states in others can activate bodily states in the self^4,9,21,32,33^. As Maister *et al*.^9^ suggested “such a ‘bodily resonance’ can afford a unique, first-person understanding of the experiences of others and is central to social processes”. These social processes include likeability, likeness, empathy and action understanding towards others among many others social behavioural features. Accordingly here we report how, by manipulating congruent visuo-motor information, we can bias body-self representation, as well as emotional perception over a plausibly enacted robot. Critically, while the subject and the robot facing at each other through a mirror were making synchronous head movements, the human subject judged the robot he/she beamed into significantly more likeable and socially closer. Control conditions whereby participants’ head movements induced unmatched movements (uncorrelated) or no movement at all (static) over the robot’s head, produced instead a reduction of the robots’social attraction. In some participants, the beaming experience was strong enough to produce a likeability score reversal, with respect to their initial preference. Moreover, the social attraction was often linked to the level of embodiment including the agency dimension as there were significant correlations between the likeability and/or closeness and agency scores. Such social changes observed after human beaming into a robot resemble the observations on affective and social changes after manipulations of body ownership^10,32,34^. Evidence has been provided that seeing an unfamiliar face being touched synchronously and congruently as one’s own face (interpersonal multisensory stimulation = IMS), induces changes in self-face recognition with incorporation of the other’s features into body self-representation^8,10,23^. In the same vein several recent studies using IMS, demonstrated that these sensory-driven adjustments in self-other boundaries can also have profound implications in term of more conceptual representation of others relative to self, including acceptance of racial and political outgroups^11,32,34^. For example, changes in embodied self- representation, like changes in ownership toward the body of an outgroup member, might reduce the negative attitude bias toward the members of this outgroup. Likewise in our study, the experimental beaming produced a body self re-location and a sense of agency i.e. of motor control over the robot independently of the type of the robot either humanoid or fiction personage. Even more, the affective feeling, especially the likeability was improved making a better acceptance of robot physical features possible. As the majority of previous studies used sensory driven manipulation to induce embodiment and changes in self recognition, our findings provide new insights showing that motor driven manipulation can induce similar effects.

A major challenge for creating robots that will interact socially with people in everyday contexts is to reduce the psychological barrier between humans and robots, ultimately promoting their acceptance. Integration of anthropomorphic details implemented into the appearance and behaviour of robots can promote social acceptance, but only up to a certain degree. Excessive similarity to humans can, in fact, trigger negative emotional responses towards robots, the so-called ‘uncanny valley’ phenomenon^35^. Here we report for the first time that when humans embody robots via sensori-motor “beaming” into the robotic bodies, this beaming experience promotes social acceptance of these robots. Further, this social acceptance is achieved in robotic bodies, humanoid or not, implying humans can embody non-human-like robots.

The major limitation of this study resides in the qualitative measurements of the embodiment sensation obtained after the beaming session in each experiment. However, even though the measurements rely upon qualitative tests and questionnaires, the findings were quite consistent in the successive experiments and also between the different robots. The selection of questionnaire items were inspired by previous studies describing significant feelings of embodiment^7,16,36,37^ into body parts^2,14,16,37^ or whole-body^3,6^ which are quite comparable to our results during robot embodiment. In the future, it would be interesting to quantify the level of embodiment in robotic bodies by measuring objective variables during the beaming session in order to monitor and to adapt on-line the feeling of incorporation into robots in particular during telepresence applications.

## Conclusions

These novel results reveal that our simple, and fast beaming procedure can produce systematic changes into the observer’s social attitude towards robots. Remarkably, the procedure can actually reverse subject’s initial preferences for one robot over another. By taking a different stance, built on a sensory-motoric embodiment approach, we have provided proof-of-concept that beaming into robots can overcome the major challenge of making them socially acceptable.

## Material and Methods

### Experiment1

#### Participants

**Figure 4:**
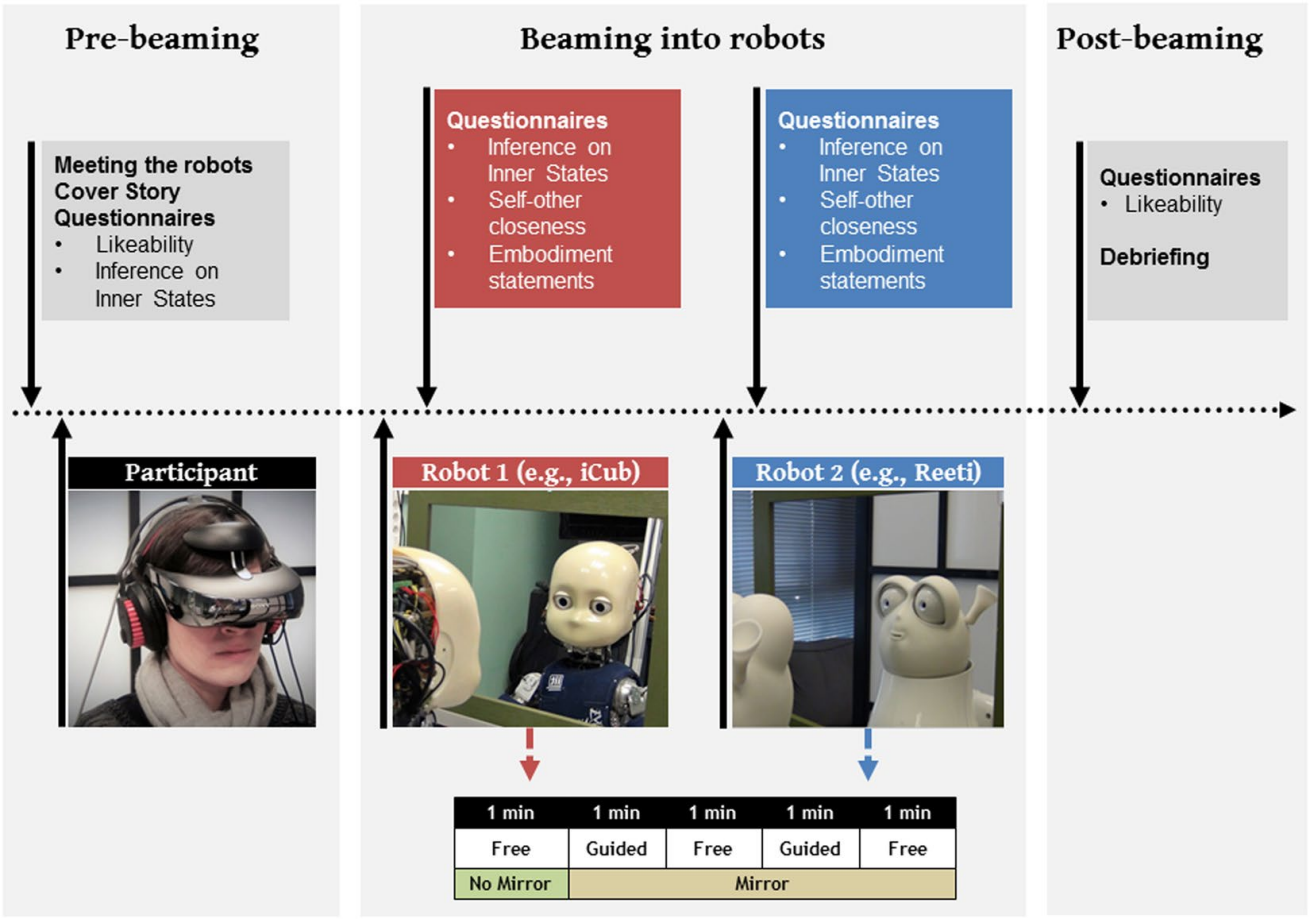
Experimental setup: Photographs of the experimental set-up showing the subject with the head-mounted camera and the robots iCub and Reeti in front of the mirror whereby the subject sees the robot face. The successive phases of the experimental procedure are sketched in the bottom panel.

A group of 16 healthy, right-handed subjects (8 males, mean age = 24.0 years SD = 2.8) participated in the experiment. Subject numerosity was established based on the effect size reported in previous works addressing the effect of multisensory illusions on self and social perception^7,38^, using the G*Power software -version 3.1.9.3^39^- to achieve a power equal or above 0.75. All of the subjects reported no history of drug abuse, no neurological and psychiatric disorders. When engaged in the experiment, the subject was naive to the actual aim of the study and gave her/his informed consent to participate to the study. The study was performed under approval (Authorization No. 10028) from the Rhône-Alpes Préfecture review board authorizing biomedical research at the Stem Cell and Brain Research Institute. The experiment was carried out in accordance with the principles of the revised Helsinki Declaration (World Medical Association, 2013). People who appear in the Fig. 4 and in the Supplementary Material (SM1) gave their informed consent for publication of identifying images in an online open-access publication.

#### Methods

Robots reeti and iCub: This study was performed with two robots: the robot iCub^40^, and the robot Reeti (Robopec, 83140 Six-Fours-les-Plages- France, http://www.reeti.fr/index.php/en/). As shown in Fig. 4, both robots had a mobile head with facial features including eyes, nose, mouth and ears. Critically, the eyes and heads of both robots were mobile. Video cameras inserted in the robot eyes allowed the robot to explore the visual environment. While the robot iCub had a humanoid structure (size of a 3 year-old child), the robot Reeti resembled a cartoon character.

Set up: The subject was sitting in the experimental room in a location several meters from the robots in order not to be visible for the robot and not to see the robot. As shown in Fig. 4, the subject wore a Head-Mounted Display (HMD) with a stereo display (SONY HMZ-3WT 3D Viewer) connected to the video cameras located in the robot eyes, and an audio helmet to isolate the subject from environmental noise. A small head motion sensor fixed on the HMD was coupled to a transducer (Polhemus Fastrak TM, 05446 Vermont, USA) located in the close proximity of the subject. Thus, the registered head motion rotation signals were transmitted to the robot via a PC computer. The Polhemus Fastrak has documented low latency (~4 ms), and the overall latency between subject’s head movement and robot’s head movement was less than100 ms^12^.Subjects were instructed to make ample but relatively slow head movements, which minimized any subjective perception of latency. To verify this last point, all subjects were asked about the perception of synchrony during the debriefing time. With this set-up the robot could reproduce the subject’s head displacements with a delay not detectable by the subject. This system called Super Wizard of Oz, SWoOZ was initially made to investigate the movement parameters critical for a realistic social interaction between human and robot^12,41^. A video is provided in the Supplementary Material (SM1) to illustrate the setup for subject beaming into the robot.

Procedure: The successive phases of the procedure are illustrated in Fig. 4.

Pre-beaming phase: In order to have an optimal participation, the subject was told that “the study is aimed to determine the best psychological profile for a robot to be further involved in social interactions in commercial or cultural contexts”. Then the subject was introduced to each robot (names, examples of movements) and he/she completed the two questionnaires on Likeability and Inference on Inner States, described below.

Beaming phase: Once the beaming set-up (HMD, Polhemus Fastrak head motion sensor) was completed, the subject had a 1 min habituation with the experimental condition by freely exploring the visual environment seen through the robot’s eyes. Then, a mirror was placed at a 20 cm distance in front of the robot, with the subject hence seeing the robot’s face (robot head and shoulders visible) in place of his/her own face. In this facing-the-mirror situation, the subject was told to move the head in a guided or free exploration manner. Guided exploration consisted of a 1 min guided sequence of 25 short head movements directed towards the mirror edges as verbally stated by the experimenter (e.g. upper left, lower right, middle left, etc.). The free exploration consisted of a 1 min sequence of free movements when the subject freely explored the reflections in the mirror, in particular the moving robot’s head. These phases alternated twice as detailed in Fig. 4.

Critically, the robot’s head movements could be synchronous to the subject’s head movements (Correlated condition) or the robot’s head could be static even though the subject’s head was moving (Static condition). In Static condition, the subject was encouraged to move the head in order to induce eventual synchronous head movements of the robot. This instruction was given to ensure similar head displacements in the different beaming conditions.

After the beaming experience with one robot, the subject was asked to respond carefully to questionnaires (Inference on Inner States, Self-other Closeness, Embodiment statements). Then, the second robot was tested in a similar beaming experimental session, but with a different movement condition. Hence, the first robot could be experienced in the synchronous/correlated movement condition, whereas the second robot was experienced in the static movement condition (or vice-versa). Order of presentation of the robot (iCub or Reeti) and order of the movement conditions (correlated or static) were counterbalanced across participants.

Post-beaming phase: At the end of the beaming sessions with the two robots, the subject was asked to respond to the questionnaire of Likeability towards the two robots, then during a debriefing time to report spontaneously about the whole experiment and about his/her sensations induced by the experimental beaming into the robots.

Questionnaires and dependent variables: Four questionnaires were used to quantify the degree of social acceptance and embodiment in the robot. For the Likeability, IOS and Embodiment questionnaires, the subject had to rate his/her feeling or sensation between the two extremes of a subjective scale.

- Measurement of Likeability: The Likeability for the robot was estimated while the subjects viewed pictures of the two robots. For each one the subject had to evaluate her/his Likeability on a subjective scale from 0 (not at all likeable) to 100 (very likeable). This test was presented to the subject in the Pre- and Post-beaming phases of the experiment (see Fig. 4).
- Inferences on Inner States (IIS): Based on Mitchell’s task^7,42^ the inference task was presented before and after beaming into each robot (see Fig. 4). In the Pre-beaming phase, the participant rated a set of 15 questions related to psychological traits (opinions and habits) referring to her/himself (“Do you arrive in time at meetings?”). Then, after each beaming session the same questions were rated referring to the previously embodied robot (“Does he arrives in time at meetings?”).
- Self-other closeness task: Based on the Inclusion of the Other in the Self scale (IOS, ref.^43^), this task assessed the degree of closeness for the robot. A graphic 7-point scale was made of a series of two coupled circles illustrating the self and the other (here the robot) with different degree of overlapping from distant circles (1) to coinciding circles (7) (see Fig. 2). This test was presented after each of the experimental beaming conditions (i.e., Static, Correlated).
- Embodiment statements: The subject rated her/his degree of embodiment within the robot on a subjective scale ranging from 0 (no sensation of embodiment) to 100 (highly strong sensation of embodiment). Four types of questions were presented to the subject (1) on the feeling that her/his face became the robot face (enfacement: 6 items), (2) on the feeling to be at the location of the robot (location: 5 items) and (3) on the feeling to control the robot’s motion (agency: 7 items). This test was presented after each of the experimental beaming conditions (i.e. Static, Correlated). The embodiment statements are detailed in the Supplementary Material (SM2).

Data analysis: The analysed variables were the Likeability score, the IIS score, the IOS score and the Enfacement, Location, Agency scores. The Likeability score was calculated as the percentage of the maximal score of the subjective scale (100). The IIS score was assessed by attributing the value 1 and 0 to the positive and negative responses respectively. The IOS score corresponded to the value of the degree of closeness chosen by the subject. The Embodiment rating was evaluated for each category (Enfacement, Location, Agency) by averaging the percentage of the maximal score of the subjective scale (100) obtained at each item.

Statistical analyses were realised with Statistica software package (TIBCO Software Inc., CA 94304-USA). Each variable was analysed for the normal distribution using the Kolmogorov-Smirnov test and for variance homogeneity using the Levene’s test. As all the variables distribution with the exception of the IIS score met the assumptions of normality and variance homogeneity at p > 0.05, we used statistical parametric tests, including the analysis of variance (ANOVA) for Likeability, Closeness and Embodiment scores.

For Likeability, we used a mixed repeated measures ANOVA design with two within-subject factors as Beaming Experience (Pre, Post) and Robot State (R-State: Static, Correlated) and one between subject factor Robot Type, identifying which robot was experienced as synchronous/correlated (R-Type: Reeti, iCub). For the IOS, a mixed repeated measures ANOVA with one within-subject factor, the Robot State (R-State: Static, Correlated) and one between subject factor, the Robot Type (R-Type: Reeti, iCub). For the Embodiment scores, a mixed repeated-measures ANOVA was used with two within-subject factors, the Robot State (R-State) and the Embodiment Category (enfacement, location, agency) and the between-subject factor the Robot Type (R-Type: Reeti, iCub). A Pearson correlation analysis corrected for multiple comparison (Bonferroni corrected significance at p = 0.012) was performed between the different variables Likeability, IOS, Ownership, Location and Agency. For the IIS test, scores were correlated between the Self and Other responses with a Spearman test (non Gaussian distribution of binary variables). We analysed the within-participant correlations between the ratings of the Self and the ratings of the Other (the robot) in the Static condition, and between the ratings of the Self (the subject) and the ratings of the Other (the robot) in the Correlated condition. Post-hoc specific comparisons were realised with a Bonferroni test and the significance was established at 95% of confidence interval.

### Experiment 2

#### Participants

A new group of 16 healthy, right-handed subjects (8 males, mean age = 23.80. years SD = 4.00) participated in the experiment. All of the subjects reported no history of drug abuse, nor neurological nor psychiatric disorders. When engaged in the experiment, the subject was naive to the actual aim of the study and gave her/his informed consent to participate to the study. The study was performed under approval (Authorization No. 10028) from the Rhône-Alpes Préfecture review board authorizing biomedical research at the Stem Cell and Brain Research Institute. The experiment was carried out in accordance with the principles of the revised Helsinki Declaration (World Medical Association, 2013).

#### Methods

The robots, set-up and paradigm were the same as in Experiment 1, except for the static (Static condition) beaming mode that was replaced by an uncorrelated (Uncorrelated condition) mode where the robot’s movements unmatched the subject’s movements. In the uncorrelated condition, while the subject freely moved the head in a slow, self-paced exploratory manner, the robot’s head was autonomously guided by a pre-recorded pseudo-random motion trajectory generated to roughly match the same spatio-temporal features. A set of four of such trajectories were counterbalanced across subjects. The instructions were the same and in Uncorrelated condition the subject was encouraged to move the head in order to induce eventual synchronous head movements of the robot (as in Static condition of experiment1). Statistical analyses were identical to those used for Experiment 1, with the only exception that the Uncorrelated factor level now replaced the previous Static factor level.

## Supplementary information

SM1-Video

SM1 Setup Description

SM2 Statements on embodiment

## Data Availability

The datasets generated during and/or analysed during the current study are available from the corresponding author on reasonable request.
