# Supplementary material for: Embodiment into a robot increases its acceptability
Source: Sci Rep. 2019 Jul 12;9:10083. doi: 10.1038/s41598-019-46528-7 (PMC6625983; doi:10.1038/s41598-019-46528-7)
Supplement: Supplementary file 2 — SM1 Setup Description [file 41598_2019_46528_MOESM2_ESM.docx]

SM1 of “Embodiment into a robot increases its acceptability” by J. Ventre-Dominey, G. Gibert, M. Bosse-Platiere , A. Farnè , P.F. Dominey and F. Pavani .

Title: Video of the setup

This video illustrates the setup for subject beaming into the robot.

See Video in uploaded file : Ventre Dominey-SM1-MOVIE.MP4
