# Supplementary material for: Embodiment into a robot increases its acceptability
Source: Sci Rep. 2019 Jul 12;9:10083. doi: 10.1038/s41598-019-46528-7 (PMC6625983; doi:10.1038/s41598-019-46528-7)
Supplement: Supplementary file 3 — SM2 Statements on embodiment [file 41598_2019_46528_MOESM3_ESM.docx]

SM2 of “Embodiment into a robot increases its acceptability” by J. Ventre-Dominey, G. Gibert, M. Bosse-Platiere , A. Farnè , P.F. Dominey and F. Pavani .

Title: Statements on embodiment

Statements on the illusory sensation of embodiment: Enfacement from 1 to 6- Location from 7 to 11- Agency from 12 to 18.

1. I had the feeling that the robot’s face started to resemble my face.
2. I had the feeling that the size of my head was reduced
3. I had the feeling that my skin became pale
4. I had the feeling that my nose was smaller
5. I had the feeling that my skin became rubbery
6. I had the feeling that the robot face belonged to me
7. I had the feeling that I was seeing myself in the mirror
8. I had the feeling that the robot face was my own face
9. I had the feeling that I was in the place of the robot
10. I had the feeling that I was inside the robot
11. I had the feeling that I was protected inside the robot
12. I wanted to move as a robot
13. I had the feeling that the movements of the robot head were the movements of my own head
14. I had the feeling that the movements of the robot head were reproducing my own movements
15. I had the feeling that I was controlling the robot head
16. I had the feeling that I was controlling the robot eyes
17. I had the feeling that I was able to make the robot speak
18. I had the feeling that if I was making faces, the robot would make the same faces.
